# Burden of disease in Germany attributed to ambient particulate matter pollution: Findings from the Global Burden of Disease Study 2019

**DOI:** 10.1007/s00059-024-05269-8

**Published:** 2024-09-10

**Authors:** Omar Hahad, Jos Lelieveld, Sadeer Al-Kindi, Volker H. Schmitt, Lukas Hobohm, Karsten Keller, Martin Röösli, Marin Kuntic, Andreas Daiber

**Affiliations:** 1 Department of Cardiology, Cardiology I, University Medical Center of the Johannes Gutenberg University Mainz, Mainz, Germany; 2 German Center for Cardiovascular Research (DZHK), Partner Site Rhine-Main, Mainz, Germany; 3 Atmospheric Chemistry, Max Planck Institute for Chemistry, Mainz, Germany; 4 Houston Methodist DeBakey Heart and Vascular Center, Houston, TX USA; 5 Center for Thrombosis and Hemostasis (CTH), University Medical Center of the Johannes Gutenberg University Mainz, Mainz, Germany; 6 Swiss Tropical and Public Health Institute, University of Basel, Basel, Switzerland

**Keywords:** Air pollution, Chronic noncommunicable diseases, Disability, Mortality, Global Burden of Disease Study 2019, Luftverschmutzung, Chronische nichtübertragbare Krankheiten, Behinderung, Mortalität, GBD(Global Burden of Disease)-Studie 2019

## Abstract

**Introduction:**

Ambient fine particulate matter pollution with a diameter less than 2.5 micrometers (PM_2.5_) is a significant risk factor for chronic noncommunicable diseases (NCDs), leading to a substantial disease burden, decreased quality of life, and deaths globally. This study aimed to investigate the disease and mortality burdens attributed to PM_2.5_ in Germany in 2019.

**Methods:**

Data from the Global Burden of Disease (GBD) Study 2019 were used to investigate disability-adjusted life–years (DALYs), years of life lost (YLLs), years lived with disability (YLDs), and deaths attributed to ambient PM_2.5_ pollution in Germany.

**Results:**

In 2019, ambient PM_2.5_ pollution in Germany was associated with significant health impacts, contributing to 27,040 deaths (2.82% of total deaths), 568,784 DALYs (2.09% of total DALYs), 135,725 YLDs (1.09% of total YLDs), and 433,058 YLLs (2.92% of total YLLs). The analysis further revealed that cardiometabolic and respiratory conditions, such as ischemic heart disease, stroke, chronic obstructive pulmonary disease, lung cancer, and diabetes mellitus, were the leading causes of mortality and disease burden associated with ambient PM_2.5_ pollution in Germany from 1990–2019. Comparative assessments between 1990 and 2019 underscored ambient PM_2.5_ as a consistent prominent risk factor, ranking closely with traditional factors like smoking, arterial hypertension, and alcohol use contributing to deaths, DALYs, YLDs, and YLLs.

**Conclusion:**

Ambient PM_2.5_ pollution is one of the major health risk factors contributing significantly to the burden of disease and mortality in Germany, emphasizing the urgent need for targeted interventions to address its substantial contribution to chronic NCDs.

## Abstract (de)

**Einleitung:**

Die Umweltbelastung durch Feinstaub mit einem Durchmesser < 2,5 Mikrometer (PM_2,5_) ist ein wesentlicher Risikofaktor für chronische nichtübertragbare Krankheiten (NCDs) und führt weltweit zu einer erheblichen Krankheitslast, zu verminderter Lebensqualität und zu Todesfällen. Ziel dieser Studie war es, die Krankheits- und Mortalitätslast durch PM_2,5_ in Deutschland im Jahr 2019 zu untersuchen.

**Methoden:**

Daten der GBD(Global Burden of Disease)-Studie 2019 wurden verwendet, um DALYs („disability-adjusted life years“), die YLLs („years of life lost“), YLDs („years lived with disability“) und die Todesfälle zu ermitteln, die auf die PM_2,5_-Belastung in Deutschland zurückzuführen sind.

**Ergebnisse:**

Im Jahr 2019 war die PM_2,5_-Belastung in Deutschland mit erheblichen gesundheitlichen Auswirkungen verbunden. Sie trug zu 27.040 Todesfällen (2,82 % der Todesfälle insgesamt), 568.784 DALYs (2,09 % aller DALYs), 135.725 YLDs (1,09 % aller YLDs) und 433.058 YLLs (2,92 % aller YLLs) bei. Darüber hinaus ergab die Analyse, dass kardiometabolische und respiratorische Erkrankungen, wie etwa ischämische Herzerkrankung, Schlaganfall, chronisch-obstruktive Atemwegserkrankung, Lungenkrebs und Diabetes, die Hauptursachen für Mortalität und Krankheitslast im Zusammenhang mit der PM_2,5_-Belastung in Deutschland von 1990–2019 darstellten. Vergleichende Bewertungen zwischen 1990 und 2019 verdeutlichten, dass die PM_2,5_-Belastung durchgehend ein prominenter Risikofaktor war, der eng mit traditionellen Faktoren, wie Rauchen, arterieller Hypertonie und Alkoholkonsum, zusammenhing und zur Mortalität sowie zu DALYs, YLDs und YLLs beitrug.

**Fazit:**

Die PM_2,5_-Umweltbelastung ist einer der wesentlichen Risikofaktoren, der erheblich zur Krankheits- und Mortalitätslast in Deutschland beiträgt. Dies unterstreicht die dringende Notwendigkeit gezielter Interventionen, um den substanziellen Beitrag dieses Faktors zu chronischen NCDs anzugehen.

## Introduction

The global disease landscape has transitioned from communicable, maternal, perinatal, and nutritional causes to noncommunicable diseases (NCDs) characterized by atherosclerotic or metabolic sequelae, notably ischemic heart disease, arterial hypertension, and diabetes mellitus, as reported by the World Health Organization (WHO)—Global Health Observatory and the Global Burden of Disease (GBD) Study [1, 2]. In 2010, prominent risk factors for global mortality encompassed tobacco smoking, arterial hypertension, ischemic heart disease, and cerebrovascular disease, collectively constituting approximately 55% of global deaths [3, 4]. The GBD Study 2019 identified arterial hypertension (resulting in 10.8 million global deaths; 19.2% of total deaths) and tobacco smoking (causing 8.7 million global deaths; 15.4% of all deaths) as the leading risk factors of global mortality [5].

While historical scientific efforts primarily focused on traditional health risk factors such as diabetes, smoking, and arterial hypertension [3], the GBD Study 2019 revealed the existence of novel environmental factors fostering chronic NCDs and contributing to global mortality [5]. The Lancet Commission on pollution and health underscored degraded air quality as the preeminent environmental cause of disease and premature death globally. Diseases arising from air pollution accounted for an estimated 9 million premature deaths in 2015, surpassing the combined mortality of acquired immunodeficiency syndrome (AIDS), tuberculosis, and malaria by approximately threefold [6]. The chief contributor is ambient air pollution, reducing the global average life expectancy by about 2.9 years, surpassing the impact of traditional health risk factors like tobacco smoking (2.2 years) [7]. The WHO indicates that up to 12.6 million global deaths in 2012 were attributable to unhealthy environments [8, 9].

Recent estimations indicate that, in 2020 alone, 9 million premature deaths globally were linked to air pollution in the form of fine particulate matter (PM) [10–12]. PM pollution includes a diverse range of substances that originate from primary sources such as traffic, energy production, industrial activities, construction, fires, and waste incineration, as well as secondary formation through gas-to-particle conversion in the atmosphere. PM is typically classified by particle size, including inhalable PM (PM_10_), fine PM (PM_2.5_), and ultrafine PM (PM_0.1_), with the numbers representing the maximum diameter of the particles in micrometers [13–16]. Exposure to elevated levels of PM_2.5_ is associated with impaired vascular function, which can contribute to cardiovascular conditions such as myocardial infarction, arterial hypertension, stroke, and heart failure. The underlying mechanisms include PM_2.5_-induced inflammation, oxidative stress, and endothelial dysfunction [17].

While earlier publications predominantly focused on global or regional implications, limited attention has been paid to assessing the burden of disease in Germany arising from ambient PM_2.5_ pollution. This analysis fills this gap by investigating the burden of disease in Germany associated with ambient PM_2.5_ pollution, utilizing data from the GBD Study 2019 from the Institute for Health Metrics and Evaluation. To achieve this objective, disability-adjusted life–years (DALYs), years of life lost (YLLs), years lived with disability (YLDs), and deaths attributed to ambient PM_2.5_ pollution in Germany were accounted for.

## Methods

### Data source

This study utilized data from the GBD Study 2019, a comprehensive assessment encompassing epidemiological variables such as incidence, prevalence, deaths, YLDs, YLLs, and DALYs across 369 diseases and injuries, 286 mortality causes, and 87 risk factors in 204 countries and regions [5, 18]. The pertinent data on the burden of disease in Germany attributed to PM_2.5_ pollution were sourced from the GBD Results tool on the Institute for Health Metrics and Evaluation website (https://vizhub.healthdata.org/gbd-compare//). The GBD Study 2019 used long-term, global data of fine particulate matter (PM_2.5_) that were derived from satellite observations of aerosol optical depth, employing a physical relationship with PM_2.5_, ground-based air quality measurements in North America, Europe, and Asia, combined with atmospheric chemistry transport modeling according to the method described by van Donkelaar et al. [19]. The PM_2.5_ data, including uncertainty estimates are publicly available from the University of Washington at https://sites.wustl.edu/acag/datasets/surface-pm2-5/. Detailed information on data sources, statistical analyses, and modeling procedures can be found in the published works of the authors of the GBD 2019 Study [5, 18].

### Main input data

The computation of DALYs in the GBD Study entailed an analysis of basic epidemiological data. Information derived from reviews, subsequent meta-analyses, and publicly available sources underwent statistical processing. Estimates, contingent on data quantity and quality, were predominantly based on country-specific data sources or derived from prediction models that accounted for incomplete or qualitatively insufficient data. For Germany, official death registry data and International Classification of Diseases (ICD)-coded cause of death statistics were employed [20].

### Excess mortality calculations

Excess mortality expresses the number of deaths over a given period (e.g., a year) that would not occur without exposure. Excess deaths are estimated based on the spatial (globally gridded) and temporal distribution of PM_2.5_ and a relative risk function, which relates the exposure to excess mortality. The relative risk is estimated from the pooled hazard ratio, a function of the PM_2.5_ concentration, derived from numerous epidemiological cohort studies. The relative risk (RR) determines the attributable fraction (AF=RR−1/RR) of the age-dependent baseline mortality rates per disease category. The calculations account for the gridded population counts and age distributions and apply counterfactual pollution levels below which the risk of excess mortality from exposure to air pollution is negligible.

Pozzer et al. [21] have reviewed different methods to derive the RR and counterfactual pollution levels, including the meta-regression—Bayesian, regularized, trimmed (MR-BRT) tool applied in the GBD Study 2019. It accounts for the following disease categories that lead to early deaths: cardiovascular disease, stroke, chronic obstructive pulmonary disease, type 2 diabetes, lower respiratory infections, lung cancer, and adverse birth outcomes. Uncertainties are expressed by the 95% confidence intervals, accounting for the between-cohort study heterogeneity and the confidence intervals of the input data used in the RR calculations.

### Measures

The GBD 2019 Study outcomes for Germany are articulated in terms of deaths, YLDs, YLLs, and DALYs. DALYs encompass two integral components, YLLs and YLDs, serving as a measure of lost healthy life–years. In the context of burden of disease studies, “disability” denotes any quantifiable (percentage) deviation from optimal health status. The mortality component of YLLs is computed based on the number of deceased individuals (stratified by age, sex, and cause of death) and a globally standardized life expectancy at birth. The morbidity component of YLDs results from the prevalence (stratified by age and sex) of the health-impairing condition under scrutiny and disability weights, established uniformly for all health states considered in GBD 2019. These weights gauge the impact of diseases and injuries on health, ranging from 0 (complete health) to 1 (a state akin to death). The cumulative sum of YLLs and YLDs constitutes the DALYs [20].

### Ambient particulate matter pollution

Ambient PM_2.5_ pollution is represented by the annual average mass concentration of particles with an aerodynamic diameter less than 2.5 µm in a cubic meter of air [5]. All presented results include 95% uncertainty intervals (95% UI), which, akin to confidence intervals, encapsulate estimation-related uncertainties and also incorporate uncertainties originating from various sources, such as modeling uncertainties.

## Results

### Ambient particulate matter pollution and measures by all-causes

The assessment of ambient PM_2.5_ pollution in Germany for the year 2019 reveals a discernible impact on health measures, including mortality, DALYs, YLDs, and YLLs (Table 1). The estimated number of deaths attributed to ambient PM_2.5_ pollution was 27,040, constituting 2.82% of total deaths. The overall DALY burden was estimated at 568,784, accounting for 2.09% of Germany’s total DALYs. YLDs were estimated at 135,725, representing 1.09% of total YLDs. The estimated YLLs totaled 433,058, comprising 2.92% of the total YLLs.

**Table 1:** Deaths, disability-adjusted life–years, years lived with disability, and years of life lost attributed to ambient particulate matter pollution in Germany for the year 2019

Measure	Metric	Value	UI upper	UI lower
Deaths	Number	27,040.52	34,653.49	20,050.91
	Percent	2.82	3.59	2.09
	Rate	31.84	40.81	23.61
DALYs	Number	568,784.12	729,797.04	418,016.86
	Percent	2.09	2.67	1.54
	Rate	669.84	859.45	492.28
YLDs	Number	135,725.18	202,868.27	82,842.63
	Percent	1.09	1.49	0.74
	Rate	159.84	238.91	97.56
YLLs	Number	433,058.95	549,077.57	323,826.38
	Percent	2.92	3.72	2.19
	Rate	510.00	646.63	381.36

Age-standardized rate per 100,000

*DALYs* Disability-adjusted life–years, *YLDs* Years lived with disability, *YLLs* Years of life lost, *UI* 95% uncertainty interval

### Ambient particulate matter pollution and measures by communicable, maternal, neonatal, and nutritional diseases (CMNNDs) and noncommunicable diseases (NCDs)

Deaths attributed to CMNNDs resulting from ambient PM_2.5_ pollution were estimated at 1405, accounting for 4.18% of total CMNND-related deaths (Table 2). NCDs contributed 25,635 deaths, representing 2.89% of the overall NCD-related mortality. DALYs associated with NCDs reached 546,040, comprising 2.25% of the total DALYs attributed to NCDs. In the case of CMNNDs, the DALY burden was estimated at 22,744, constituting 2.37% of the total DALYs assigned to CMNNDs. YLDs due to NCDs were estimated at 135,722, accounting for 1.23% of total YLDs attributed to NCDs. CMNNDs contributed to a smaller YLD burden of 240.42 (0.06% of total YLDs related to CMNNDs). YLLs due to CMNNDs were estimated at 22,529, representing 4.26% of the total YLLs assigned to CMNNDs. For NCDs, the YLLs were 410,991, constituting 3.08% of the total YLLs attributed to NCDs.

**Table 2:** Deaths, disability-adjusted life–years, years lived with disability, and years of life lost due to communicable, maternal, neonatal, and nutritional diseases and noncommunicable diseases attributed to ambient particulate matter pollution in Germany for the year 2019

Measure	Cause	Metric	Value	UI upper	UI lower
Deaths	CMNNDs	Number	1405.33	2287.12	805.61
		Percent	4.18	6.74	2.41
		Rate	1.66	2.69	0.95
	NCDs	Number	25,635.19	32,830.20	18,875.12
		Percent	2.89	3.72	2.14
		Rate	30.19	38.66	22.23
DALYs	NCDs	Number	546,040.12	705,355.94	399,202.62
		Percent	2.25	2.91	1.65
		Rate	643.05	830.67	470.13
	CMNNDs	Number	22,744.00	36,356.67	12,505.22
		Percent	2.37	3.80	1.29
		Rate	26.78	42.82	14.73
YLDs	NCDs	Number	135,722.72	202,929.46	83,037.40
		Percent	1.23	1.69	0.83
		Rate	159.84	238.98	97.79
	CMNNDs	Number	240.42	430.45	125.35
		Percent	0.06	0.09	0.03
		Rate	0.28	0.28	0.28
YLLs	CMNNDs	Number	22,529.44	36,047.92	12,331.31
		Percent	4.26	6.87	2.39
		Rate	26.53	42.45	14.52
	NCDs	Number	410,991.32	523,498.37	306,518.92
		Percent	3.08	3.08	3.08
		Rate	484.01	484.01	484.01

Age-standardized rate per 100,000

*DALYs* Disability-adjusted life–years, *YLDs* Years lived with disability, *YLLs* Years of life lost, *CMNNDs* Communicable, maternal, neonatal, and nutritional diseases, *NCDs* Noncommunicable diseases, *UI* 95% uncertainty interval

### Ranking of causes

**Fig. 1:**
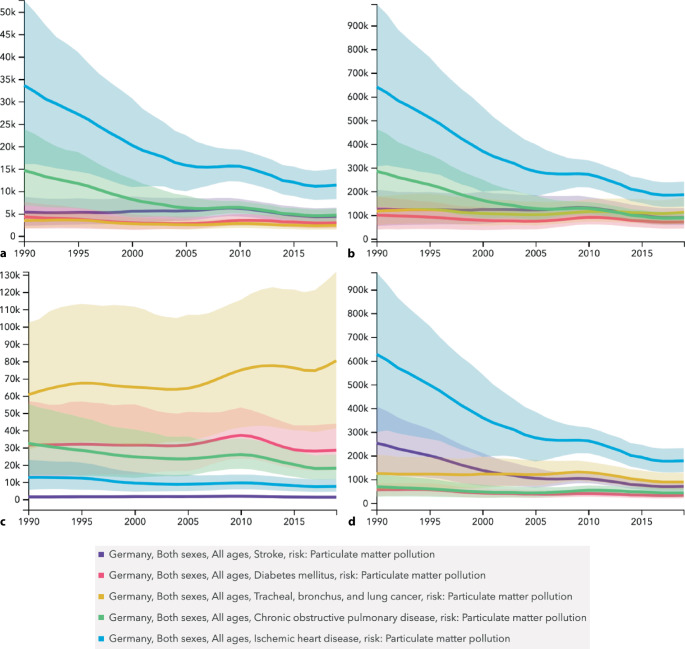
Time trend (1990–2019) for numbers (including uncertainty interval) of deaths (**a**), DALYs (**b**), YLDs (**c**), and YLLs (**d**) caused by stroke, diabetes mellitus, tracheal, bronchus, and lung cancer, chronic obstructive pulmonary disease, and ischemic heart disease attributed to ambient particulate matter pollution in Germany

**Fig. 2:**
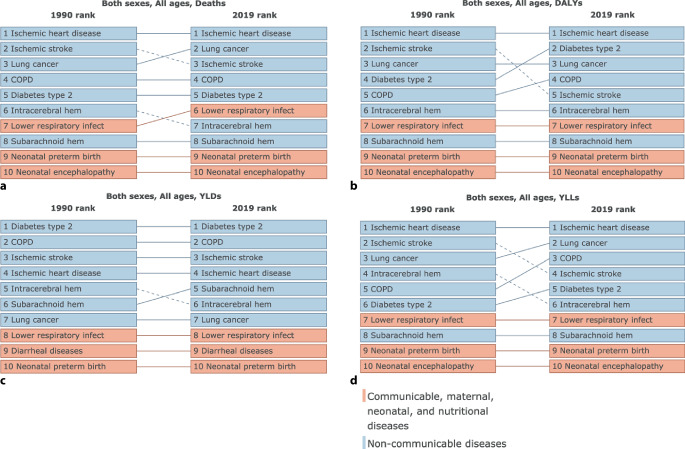
The top ten causes for number of deaths (**a**), disability-adjusted life–years (DALYs, **b**), years lived with disability (YLDs, **c**), and years of life lost (YLLs, **d**) attributed to ambient particulate matter pollution in Germany comparing the years 1990 and 2019. *COPD* Chronic obstructive pulmonary disease

Cardiometabolic and respiratory ailments, including ischemic heart disease, stroke, chronic obstructive pulmonary disease, lung cancer, and diabetes mellitus, emerged as the primary causes of number of deaths, DALYs, YLDs, and YLLs associated with ambient PM_2.5_ pollution in Germany spanning the years 1990–2019 (Fig. 1 and 2).

Notably, deaths attributed to PM_2.5_ decreased from 1990 to 2019, primarily due to a reduction in ischemic heart disease-related deaths.

### Ranking of risks

**Fig. 3:**
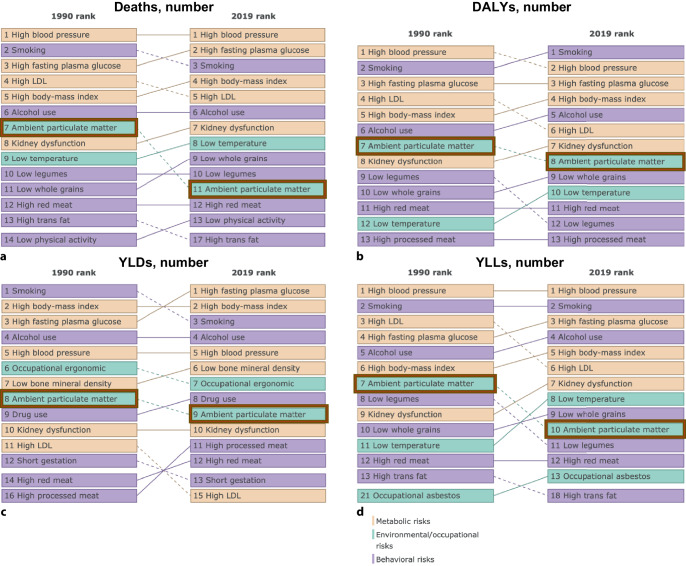
The most important risks for number of deaths (**a**), disability-adjusted life–years (DALYs, **b**), years lived with disability (YLDs, **c**), and years of life lost (YLLs, **d**) in Germany comparing the years 1990 and 2019. *LDL* Low-density lipoprotein

Comparing the years 1990 and 2019, ambient PM_2.5_ emerged as a prominent risk factor, consistently ranking among the top contributors to the number of deaths, DALYs, YLDs, and YLLs (Fig. 3). Notably, its impact remains significant over this period, with only minor variations observed ranking closely with traditional risk factors like smoking, high blood pressure, and alcohol use.

## Discussion

The assessment of ambient PM_2.5_ pollution in Germany in 2019 underscores a substantial and discernible impact on various health outcomes, including mortality, reflecting the intricate and important interplay between environmental factors and the national disease burden. The estimated 27,040 deaths attributed to ambient PM_2.5_ pollution, constituting 2.82% of the total, indicate a significant contribution to the overall mortality. The burden of disease, as measured by DALYs, amounts to 568,784, representing 2.09% of Germany’s total DALYs. This multifaceted metric encapsulates not only mortality but also YLDs and YLLs. The distinction of disease types reveals the diverse impacts of exposure to PM_2.5_ pollution. Deaths attributed to CMNNDs represent 4.18%, a significant proportion of total CMNND-related deaths, highlighting the trend in health risk factors in the past decades—from infectious diseases to chronic NCDs. Cardiometabolic and respiratory diseases, including ischemic heart disease, stroke, chronic obstructive pulmonary disease, lung cancer, and diabetes mellitus, emerge as the predominant causes of death, DALYs, YLDs, and YLLs associated with ambient PM_2.5_ pollution in Germany from 1990–2019. These results draw attention to the consistent role of ambient PM_2.5_ as a major health risk factor since 1990, even though exposure has decreased due to air quality control measures. Its persistent position among the top contributors to deaths, DALYs, YLDs, and YLLs positions it alongside traditional risk factors like smoking, high blood pressure, and alcohol use. This continuity emphasizes the need for comprehensive strategies addressing environmental exposures in tandem with established health risk factors to effectively mitigate the overall burden of disease. In conclusion, the comprehensive analysis presented here not only delineates the specific impacts of ambient PM_2.5_ pollution on health measures in Germany but also situates these findings within the broader context of global health trends, underscoring the persistent significance of environmental factors in shaping public health outcomes.

According to the European Environment Agency (EEA), air pollution is the largest environmental health risk factor in Europe. The number of deaths attributable to PM_2.5_ in Germany was estimated at 28,900 in 2020. In that year, Germany ranked among the countries with the highest number of deaths caused by PM_2.5_ particulate matter across the European Union [3]. According to the EEA, less than 1% of the EU urban population is exposed to PM_2.5_ concentrations above EU standards (25 µg/m^3^), whereas 97% are exposed to PM_2.5_ levels that exceed the new WHO guidelines from 2021 [22] (annual average concentrations of PM_2.5_ should not exceed 5 µg/m^3^, while 24 h average exposures should not exceed 15 µg/m^3^ more than 3–4 days per year) [23]. Despite the recent national downward PM_2.5_ trend in Germany, the German Environment Agency (UBA) affirms that during the years from 2010–2021, almost the entire population of Germany was exposed to PM_2.5_ concentrations above the WHO guideline level (99.97% in 2021) [24]. According to the UBA, there has been a substantial reduction in PM_2.5_ emissions in Germany (from 0.20 million tonnes in 1995 to 0.08 million tonnes in 2022, equal to a reduction of 57.8%) since 1995 [25], which may also explain the decreasing trend in deaths attributable to PM_2.5_ in the current analysis. However, due to a more pronounced decrease in total PM emissions, the relative proportion of PM_2.5_ within the total PM has significantly risen over the years [25]. Complying with the WHO guidelines is of special importance as revealed by a recent analysis of large population-based cohort studies from Canada, United States, and Europe [10]. The authors found associations down to the lowest observed levels. In particular, an analysis of 7.1 million adults in Canada, representing a global low-exposure environment, found a supralinear concentration–response relationship between ambient PM_2.5_ and mortality, particularly at very low concentrations (< 5 μg/m^3^), which is associated with an additional 1.5 million annual deaths globally attributable to ambient PM_2.5_ compared to prior estimates [26]. However, population-weighted PM_2.5_ levels in Germany were significantly lower in 2021 than in 2010. In 2021, the annual average population-weighted PM_2.5_ exposure was 9.3 µg/m^3^. This is 42% less than in 2010. Declining emissions from stationary sources (e.g., power plants, waste incineration, domestic fuel burning, and industrial facilities) and measures taken in the transportation sector are responsible for the decrease in exposure [27, 28]. The importance of phasing out fossil fuels is highlighted by a recent study, suggesting that an estimated 5.13 million (95% confidence interval 3.63–6.32) excess deaths per year globally are attributable to ambient air pollution (including PM_2.5_ and ozone) from fossil fuel use and, thus, could potentially be avoided by replacing fossil fuels by clean, renewable energy sources, which represents 82% of the maximum number of air pollution-related deaths that could be averted by controlling all anthropogenic emissions [12]. Of note, it was calculated that premature mortality-related costs attributed to PM_2.5_ in Germany in the year 2018 amounted to approximately 98 billion euros, with a range spanning from 25 to 124 billion euros [29].

While the GBD findings provide valuable insights, acknowledging its strengths, limitations should also be discussed. Notable critique includes simplification and generalization, challenges in exposure assessment, limited temporal resolution, the assumption of linearity, incomplete consideration of confounding factors, and possibly limited representation of the health effects on vulnerable populations. While the GBD Study has significantly contributed to global health policies, ongoing research efforts and improvements in modeling techniques are imperative to address these criticisms and enhance the precision of estimating the health impacts associated with ambient PM_2.5_ exposure. It needs to emphasize that the GBD assessment is based on assumptions and subject to inherent uncertainties. A recent systematic analysis of air pollution health risk assessment in Switzerland found that the exposure assessment method and the choice of the counterfactual and concentration–response function mostly affect the estimated impact [30]. For instance, using a PM_2.5_ summary estimate of European cohorts from the project ELAPSE, recommended by the European Respiratory Society and International Society for Environmental Epidemiology (ERS-ISEE [31]), resulted in 2240 deaths attributable to ambient PM in Switzerland, whereas the GBD estimated only 1374 deaths [32]. In accordance, a recent study from Germany analyzing the years 2010–2018 underscores the distinct variations in outcomes when compared to other established frameworks, notably the GBD methodology [33]. Such insights are pivotal in advancing the scientific discourse surrounding the attribution of disease burden to ambient PM_2.5_ pollution and in fostering a nuanced interpretation of the associated health risks within the German context. Furthermore, the analysis focuses on ambient PM_2.5_ pollution, and the specific characteristics of the PM_2.5_ (e.g., composition, sources) may offer nuanced insights. Also, the GBD Study does not include data on regional differences in Germany.

Taken together, our findings highlight the significant health impacts of ambient PM_2.5_ pollution in Germany and the prominence in chronic NCDs. The call for targeted interventions to address this environmental peril is underscored by the robust analysis and highlights the imperative for ongoing efforts in environmental health to curb the national disease burden.

### Key messages.

- The global disease landscape has evolved from communicable to noncommunicable diseases (NCDs), with a focus on ischemic heart disease, arterial hypertension, and diabetes mellitus.
- The Global Burden of Disease (GBD) Study 2019 highlighted novel environmental factors contributing to chronic NCDs, with chemical pollution, particularly ambient air pollution, surpassing the combined mortality of AIDS, tuberculosis, and malaria threefold.
- Ambient PM_2.5_ pollution in Germany in 2019 was linked to 27,040 deaths (2.82% of total deaths) and significant disability-adjusted life–years.
- Cardiometabolic and respiratory ailments, including ischemic heart disease, stroke, and lung cancer, were the primary causes of deaths, disability-adjusted life–years (DALYs), years of life lost (YLLs), years lived with disability (YLDs) associated with ambient PM_2.5_ pollution from 1990–2019.
- Ambient PM_2.5_ pollution consistently ranked among the top contributors to deaths, DALYs, YLLs, and YLDs globally from 1990–2019, highlighting its persistent impact alongside traditional risk factors.

## Data Availability

The publicly available data used in this analysis were obtained from the Global Burden of Disease (GBD) Study 2019 results tool from the Institute for Health Metrics and Evaluation website (https://vizhub.healthdata.org/gbd-compare//).

## Declarations

For this article no studies with human participants or animals were performed by any of the authors. All studies mentioned were in accordance with the ethical standards indicated in each case.
